# Hepatitis B Vaccination in Pregnancy in the United States

**DOI:** 10.3390/vaccines1020167

**Published:** 2013-05-08

**Authors:** Robert D. Stewart, Jeanne S. Sheffield

**Affiliations:** Department of Obstetrics & Gynecology, University of Texas Southwestern Medical Center, 5323 Harry Hines Blvd., Dallas, TX 75390, USA

**Keywords:** hepatitis B vaccine, immunization, pregnancy

## Abstract

Hepatitis B remains a significant health issue worldwide, and contributes significantly to the incidence of cirrhosis and hepatocellular carcinoma. Widespread adoption of hepatitis B vaccination strategies has lead to significant declines in acute hepatitis B infections. Current recommendations for vaccination in the non-pregnant population include vaccinating all persons found to have risk-factors for disease acquisition. Hepatitis B virus is known to occur through vertical transmission or early childhood transmission, and strategies to decrease transmission include avoidance of exposure, avoidance of high-risk behaviors, universal screening of women during pregnancy, and active and passive immunization. It is currently recommended that all pregnant women undergo screening for hepatitis B virus at presentation for prenatal care. Those who engage in high-risk behavior should be re-screened at presentation for delivery. Studies have demonstrated the safety and efficacy of the hepatitis B vaccine in pregnancy, and its use is an important component in prevention of disease acquisition. Pregnant women in the United States who are found to be at risk for disease acquisition should be specifically targeted for vaccination.

## 1. Introduction

Hepatitis B virus (HBV) is a double-stranded DNA virus of the *Hepadnaviridae* family. HBV causes infection worldwide and is endemic in Eastern Europe, the Middle East, Africa, Central and Southeast Asia, China and certain areas of South America with prevalence rates as high as 5–20%. The World Health Organization estimates that greater than two billion people worldwide are infected with HBV; 360 million have chronic infection and are at high risk for hepatocellular carcinoma and cirrhosis of the liver [1]. Approximately 600,000 deaths occur worldwide from HBV-related diseases [2]. In 2010, the Centers for Disease Control and Prevention estimated that 35,000 acute hepatitis B infections occurred in the United States, a 29 percent decrease from 2006 [3]. This decline, mirrored in most developed countries, is attributed to the widespread adoption of hepatitis B vaccination strategies.

Hepatitis B virus is transmitted by percutaneous or mucosal exposure to the blood or body fluids of infected individuals. In countries where HBV is endemic, vertical transmission from an infected mother to child peripartum or person-to-person transmission in early childhood is most common. In low prevalence countries, HBV transmission occurs more commonly via sexual transmission or sharing contaminated needles, though vertical transmission does occur. The majority of adults who become infected with HBV will eliminate the virus—only 2–8% will develop chronic hepatitis B. The converse is true for younger age groups. Chronic HBV infection develops in 80–90% of infants infected perinatally and 30–50% of children infected before the age of six [4,5].

Maternal hepatitis B infection during pregnancy does not increase maternal morbidity and mortality; in fact, it is often asymptomatic and found only on routine prenatal screening. In the absence of HBV immunoprophylaxis, 10–20% of women positive for hepatitis B surface antigen (HBsAg) transmit to their infant—this increases to almost 90% if the mother is seropositive for HBsAg and hepatitis B_e_ antigen (HBeAg) or if she develops acute HBV in the third trimester. This transmission risk decreases dramatically in the setting of universal HBV screening prenatally, immunoprophylaxis given to infants born to HBV infected mothers and, finally, hepatitis B vaccine administered both to high risk mothers and to all newborn infants.

## 2. Hepatitis B Vaccine

In 1970, Krugman, Giles, and Hammond first performed active immunization against HBV by using heated infective human serum. This serum was found to prevent or modify hepatitis B in 69% of children who were challenged [6]. Following this, several vaccines were developed and extensively tested. Initial placebo-controlled studies of an inactivated hepatitis B vaccine were conducted in men who have sex with men (MSM), due to the historically high attack rates of hepatitis B in this group. These studies demonstrated that immunization reduced the incidence of hepatitis B among this high-risk group by 90–95% [7]. This inactivated vaccine was similarly found to protect health care workers with frequent exposure to blood [8]. 

In 1982, the first hepatitis B vaccine was licensed in the United States. It was a subunit vaccine that contained 22-nm HBsAg particles that were made from the plasma of chronic HBsAg carriers. Contemporary vaccines use HBsAg that is produced by recombinant DNA technology [9]. Currently two single antigen recombinant vaccines are available in the United States: *Recombivax HB* (Merck) and *Engerix-B* (GlaxoSmithKline Biologicals). A combination vaccine, *Twinrix* (GlaxoSmithKline), containing antigens to both hepatitis A and hepatitis B is also available, and is recommended for persons ≥18 years of age who are at risk for both hepatitis A and hepatitis B infections. Two combination vaccines are also available for use in children: *Comvax* (Merck), a combined hepatitis B and Haemophilus influenzae type b (Hib) conjugate vaccine, and *Pediarix* (GlaxoSmithKline), a combined hepatitis B, diphtheria, tetanus, acellular pertussis (DTaP), and inactivated poliovirus (IPV) vaccine.

Since 1982, a comprehensive strategy for the elimination of hepatitis B in the United States has evolved. The original strategy simply targeted high-risk groups for transmission. This was subsequently shown to have a suboptimal impact on the incidence of the disease [10]. The current strategy against HBV infection includes universal vaccination of infants beginning at birth, prevention of perinatal HBV infection, routine vaccination of previously unvaccinated children and adolescents, and vaccination of previously unvaccinated adults at risk for disease acquisition [9]. In 2006, the Advisory Committee on Immunization Practices updated recommendations to increase hepatitis B vaccination coverage among adults [9,11]. Current recommendations include vaccination of: all infants beginning at birth, all children <19 years of age who have not previously been vaccinated, sexual partners of HBsAg positive persons, sexually active persons who are not in a long-term, mutually monogamous relationship, persons seeking evaluation or treatment for sexually transmitted diseases, men who have sex with men, injection drug users, susceptible household contacts of HBsAg positive persons, health care and public safety workers at risk for exposure to blood or blood-contaminated body fluids, persons with end-stage renal disease, residents and staff of facilities for developmentally disabled persons, travelers to regions with intermediate or high rates of endemic HBV, persons with chronic liver disease, persons with HIV infection, unvaccinated persons with diabetes mellitus age 19 to 59, and all other persons seeking protection from HBV [11]. Considering the last indication to receive vaccination, no specific risk factor must be identified for a person to receive vaccination. 

Current recommendations also include guidelines for universal vaccination in certain health care facilities known to care for a high proportion of hepatitis B infected persons. These facilities include: sexually transmitted disease treatment facilities, HIV testing and treatment facilities, drug abuse treatment and prevention facilities, health care settings targeting injection drug users, correctional facilities, facilities targeting services to men who have sex with men, chronic hemodialysis facilities, and institutions and nonresidential day care facilities for developmentally disabled persons [11]. 

Hepatitis B vaccination consists of three intramuscular injections in the deltoid muscle. The three-dose vaccine is administered at 0, 1, and 6 months. According to the ACIP, the minimum interval between the first and second dose is four weeks, and between the first and third dose of 16 weeks, in order to obtain an optimal immune response [11]. Approximately 30–55% percent of healthy adults ≤40 years of age will have a protective antibody response after the first dose, 75% after the second dose, and >90% after the third dose [11]. Accelerated vaccine schedules at 0, 1, and 4 months or 0, 2, and four months, have also been shown to produce similar rates of seroprotection [12]. Serologic testing for immunity, regardless of the vaccination schedule chosen, is not required after routine vaccination. However, it may be recommended in situations in which knowledge of immune status may affect medical management [11].

## 3. Hepatitis B Vaccination for Pregnant Women

Pregnancy is a unique circumstance in which screening and intervention can be utilized to impact newborn health. Similarly, pregnancy may be a time in which previously unvaccinated women present to the healthcare system, and therefore provides an opportunity for intervention on behalf of the health of the mother. To this end, the American College of Obstetricians and Gynecologists (ACOG) as well as the Centers for Disease Control and Prevention (CDC) recommend prenatal screening of all pregnant women for HBV [9,13]. Pregnant women should have a HBsAg drawn at presentation for prenatal care. Furthermore, pregnant women who undertake high-risk behavior for disease acquisition or who are not previously screened should undergo HBsAg screening when she presents for delivery. According to ACOG, pregnant women who are HBsAg negative and who are at risk for HBV infection should be specifically targeted for vaccination [9,13]. High risk women include having more than one sex partner during the previous six months, been evaluated or treated for a sexually transmitted infection, recent or recurrent injection drug use, or having an HBsAg positive sexual partner [9]. Women who are found to require vaccination for hepatitis A, based on current recommendations, as well as hepatitis B may receive a combination vaccine (Twinrix), which contains antigens to both hepatitis A and hepatitis B [13].

There is currently limited data on the use of hepatitis B vaccine in pregnancy. That being said, the American College of Obstetricians and Gynecologists, as well as the Centers for Disease Control and Prevention do not consider pregnancy a contraindication [9,13]. In fact, as mentioned above, vaccination is recommended in pregnancy in specific circumstances. The efficacy of the hepatitis B vaccine in pregnancy has been shown to be similar to the non-pregnant population [14,15]. Grosheide and colleagues examined the seroprotection conversion rates of 16 pregnant women compared to 57 non-pregnant women when the hepatitis B vaccine was given for post-exposure prophylaxis. After six months, all women who received the vaccine had protective anti-HBs levels [14]. Overall, seroconversion rates of 92–94% have been demonstrated in pregnant women [15]. Factors shown to decrease the efficacy of the hepatitis B vaccine in pregnancy include maternal obesity, advancing age, and tobacco smoking [16,17]. Despite this, the vaccine should be provided to all pregnant women at risk for disease acquisition during pregnancy.

The safety of the hepatitis B vaccine has been demonstrated in multiple studies [14,15,18,19]. In one cohort, the infants of ten women who received the plasma derived hepatitis B vaccine during the first trimester of pregnancy were followed. No congenital abnormalities were identified at delivery, and all children were physically and developmentally normal at two and 12 months [19]. Studies that have evaluated the safety of the recombinant vaccine have similarly determined that the vaccine is safe in pregnancy [14,15]. In a cohort of 16 women exposed to recombinant vaccine after in vitro fertilization, one woman had a miscarriage two days after vaccination, and one was lost to follow-up. The remaining 14 women subsequently delivered 19 healthy infants, all of who were developmentally normal at 22 months [15]. 

A critical component of hepatitis B management in pregnancy is proper immunoprophylaxis being provided to the infant after birth. Currently, both ACOG and the CDC recommend that all infants receive the hepatitis B vaccine series as part of the recommended childhood immunization schedule [9,13]. Infants born to mothers who are known to be carriers of hepatitis B, or whose status is unknown, should receive both the hepatitis B vaccine series, as well as passive prophylaxis with hepatitis B immune globulin (HBIG) [9,13]. 

The current recommended vaccination schedule in the non-pregnant population includes vaccination with the three doses of the recombinant hepatitis B vaccine at 0, 1, and 6 months. Recent studies have demonstrated that accelerated vaccination schedules of 0, 1, and 4 months are as immunogenic as the standard dosing schedule [12]. Within the pregnant population, the traditional vaccination schedule of 0, 1, and 6 months is difficult to complete in the limited time of gestation prior to delivery. After delivery, it is expected that compliance with completion of the vaccination schedule will decrease. It would therefore be ideal to complete the vaccination schedule prior to delivery, while the woman is continuing to receive regular prenatal care. In a cohort of 200 pregnant women, the recombinant hepatitis B vaccine was given at an accelerated schedule of 0, 1, and 4 months [17]. Of the 200 women enrolled, 84% completed the 3-dose vaccine schedule. After three doses, 90% of women demonstrated seroconversion, rates similar to those demonstrated in the non-pregnant population undergoing the traditional schedule of vaccination. This accelerated vaccination schedule has been shown to be an effective means of completing hepatitis B vaccination during the course of pregnancy [17]. Serologic testing for immunity is not required following this accelerated schedule of vaccination. This provides another effective tool in decreasing HBV infection.

When discussing the issue of hepatitis B vaccination in pregnancy, maternal acceptance of the vaccine series must be addressed, regardless of the vaccine schedule recommended. In a recent Chinese survey of pregnant women, maternal uptake of hepatitis B vaccine was found to be 33% [20]. The factors associated with maternal acceptance of the vaccine included employment as a healthcare worker, higher education, higher family income, routine medical checkups, and premarital checkups. Based on this, they concluded that the public lacked sufficient knowledge of hepatitis B infection. This was corroborated in a follow-up study in which a questionnaire regarding hepatitis B infection was administered to pregnant Chinese women, and indicated that misconceptions regarding the virus remained prevalent among that population [21]. Interestingly, 87.4% of the 1,623 respondents to this survey correctly answered that hepatitis B infection can be prevented by screening and vaccination, implying that confidence in the vaccine is not a major deterrent to receiving the vaccine. It is clear that maternal knowledge regarding the virus itself is an essential component of vaccine uptake. According to the CDC, implementation strategies for the hepatitis B vaccine include providing information to all adults regarding the health benefits of the hepatitis B vaccination [11]. Pregnant women for whom the hepatitis B vaccine is recommended should equally be educated on the importance of receiving the vaccination, including the benefits to both maternal and infant health.

## 4. Conclusions

Hepatitis B virus infection is a world-wide public health crisis, both acutely and more importantly, from sequelae in chronically infected individuals. Transmission occurs via vertical transmission or early childhood infection in the majority of cases in countries where HBV is endemic. Effective strategies to decrease this transmission risk include prevention of blood and body fluid exposure, avoidance of high-risk behaviors, universal screening of women during pregnancy, and active and passive immunization. The hepatitis B vaccine has been shown to be both safe and effective in pregnant women, yet is not routinely given to high-risk women in most countries, including developed nations. Education and vaccine availability need to be improved and further investigation into booster dosing and the effects of different vaccine formulations and intervals needs to be encouraged.
